# Current Status and Future Perspectives of Superior Mesenteric Artery Dissection in Robotic Pancreaticoduodenectomy: A Scoping Review of Technical Variations in the Robotic Era

**DOI:** 10.3390/jcm14176084

**Published:** 2025-08-28

**Authors:** Yosuke Inoue, Kosuke Kobayashi, Tomotaka Kato, Sho Kiritani, Atsushi Oba, Yoshihiro Ono, Hiromichi Ito, Yu Takahashi

**Affiliations:** 1 Division of Hepatobiliary and Pancreatic Surgery, Cancer Institute Hospital, Japanese Foundation for Cancer Research, Tokyo 135-8550, Japan; kosuke.kobayashi@jfcr.or.jp (K.K.); atsushi.oba@jfcr.or.jp (A.O.); yoshihiro.ono@jfcr.or.jp (Y.O.); hiromichi.ito@jfcr.or.jp (H.I.); yu.takahashi@jfcr.or.jp (Y.T.); 2 Department of Surgery, Ohta Nishinouchi Hospital, Fukushima 963-8558, Japan; tomotakakato0416@icloud.com; 3 Hepato-Biliary-Pancreatic Surgery Division, Department of Surgery, Graduate School of Medicine, The University of Tokyo, Tokyo 113-8655, Japan; sho.kiritani@jfcr.or.jp

**Keywords:** pancreaticoduodenectomy, robotic surgery, superior mesenteric artery

## Abstract

**Background**: Dissection around the superior mesenteric artery (SMA) is a key step for local clearance of periampullary cancers in pancreaticoduodenectomy (PD). Since the 2000s, SMA-first approaches have gained popularity in open surgery to allow early vascular control and resectability assessment. With the rise of robotic pancreaticoduodenectomy (RPD), various SMA dissection techniques have been adapted to the robotic setting. **Objective**: To map current evidence on SMA dissection techniques in RPD and summarize technical variations. **Eligibility Criteria and Sources of Evidence**: A PubMed search identified 116 records. After title and abstract screening and full-text review, 27 studies focusing on SMA dissection for periampullary tumors in RPD with sufficient technical detail were included. Studies on open/laparoscopic PD, lacking technical description, or reporting duplicate techniques were excluded. **Charting Methods:** Data were charted based on the SMA approach type, surgical details, and institution. **Results:** Among the 27 included studies, multiple approaches were identified—anterior, right posterior, left posterior, uncinate, and mesenteric—each adapted to the robotic platform. Techniques varied in exposure, lymphadenectomy, and vessel control. **Conclusions:** This scoping review reveals diverse SMA dissection strategies in RPD. While technical innovation is progressing, further studies are warranted to standardize approaches and assess their oncologic and surgical outcomes.

## 1. Introduction

Pancreaticoduodenectomy (PD), commonly referred to as the Whipple procedure, represents a standard surgical strategy for treating periampullary neoplasms arising from various malignancies. Despite its widespread adoption, PD is inherently associated with substantial perioperative risks, with morbidity rates ranging from 30% to 40% and mortality rates reported between 1% and 6%, even in specialized high-volume centers. The application of minimally invasive techniques to PD has historically been limited due to technical complexity. Nevertheless, recent advancements in surgical technology and robotic platforms have facilitated the growing implementation of robotic pancreaticoduodenectomy (RPD) in clinical practice [1,2].

Oncological dissection during PD primarily targets two critical arterial systems: the superior mesenteric artery (SMA) and the celiac–hepatic arterial axis. Complete clearance of these vascular structures is essential for achieving negative surgical margins and optimizing oncological outcomes in patients undergoing PD. The SMA is the key anatomical feature of the pancreatic head, duodenum, and proximal jejunum and is frequently involved directly or indirectly in periampullary cancers. Consequently, precise and oncologically sound dissection around the SMA represents a critical component of PD to ensure effective local disease control [3]. Historically, SMA dissection and lymphadenectomy were reserved for the final phase of PD. More recently, however, the SMA-first approach has gained acceptance in open surgery, allowing for early vascular control and intraoperative assessment of resectability. In the context of open pancreaticoduodenectomy (OPD), multiple strategies and well-defined dissection techniques for SMA exposure have been established and widely adopted in surgical practice [4]. However, the optimal approach according to various situations during RPD has not yet been fully discussed in the literature. In this review article, we review the current state-of-the-art of RPD, focusing on SMA dissection and discussing the characteristics of each approach and future perspectives.

## 2. Methods

### 2.1. Search Strategy

This review was conducted in accordance with the PRISMA Extension for Scoping Reviews (PRISMA-ScR) guidelines [5]. The objective was to comprehensively map and compare the reported surgical approaches to the SMA during RPD, with a focus on technical variation, evolution, and current practice. A literature search was conducted using the PubMed database. The search strategy combined Medical Subject Headings (MeSHs) and free-text terms relevant to robotic pancreaticoduodenectomy and SMA dissection techniques. The following keywords and Boolean operators were used:

“robotic pancreaticoduodenectomy” OR “robotic pancreatoduodenectomy” AND

(“superior mesenteric artery” OR “approach” OR “right approach” OR “left approach” OR “posterior approach” OR “anterior approach” OR “mesenteric approach”)

The search was limited to articles published in English between 1 January 2010, and 31 February 2025.

### 2.2. Eligibility Criteria

Studies were included if they satisfied the following:

- Addressed technical aspects of SMA dissection during robotic pancreaticoduodenectomy.
- Included original data (case series, cohort studies, or technical reports).
- Were published in peer-reviewed English journals.

Studies were excluded if they satisfied the following:

- Focused exclusively on open or laparoscopic pancreaticoduodenectomy.
- Did not describe the SMA dissection technique in sufficient detail.
- Were editorials, commentaries, conference abstracts, or non-English articles.

### 2.3. Selection Process

Titles and abstracts were screened by two independent reviewers. Full-text review was conducted for studies meeting the inclusion criteria. Discrepancies were resolved by consensus.

### 2.4. Data Extraction and Synthesis

Data were extracted regarding the following:

- Type and name of the SMA approach (e.g., right, left, posterior, anterior).
- Procedural details and scope position.
- Technical tips regarding retraction when available.
- Robotic platform used.

A narrative synthesis was performed, organizing the findings by approach type and highlighting technical evolution and future perspectives.

### 2.5. PRISMA-ScR Compliance

A completed PRISMA-ScR checklist is provided in the Supplementary Material (Table S1).

## 3. Results

A total of 116 records were identified through PubMed database searching. After removing duplicates, 116 articles remained and were screened based on their titles and abstracts. Of these, 79 records were excluded due to irrelevance or insufficient technical focus.

Subsequently, 37 full-text articles, along with any available supplemental videos, were assessed for eligibility. Among them, 10 studies were excluded for the following reasons:

- Exclusive focus on open or laparoscopic pancreaticoduodenectomy (*n* = 1);
- Insufficient description of the SMA dissection technique (*n* = 7);
- Overlapping reports from the same institution within the same timeframe (*n* = 2)

As a result, 27 studies were included in the final qualitative synthesis. The PRISMA-ScR flow diagram summarizing the selection process is presented in Figure S1.

## 4. Types of SMA Approach During PD

The concept of SMA approaches was originally developed in relation to OPD, where the SMA-first strategy was advocated to achieve reduced intraoperative blood loss, earlier determination of resectability, and more precise control over the extent of dissection. Sanjay et al. comprehensively summarized the various SMA approaches used in open PD, categorizing them into six types: superior, anterior, posterior, left posterior, right/medial uncinate (right-sided SMA approach beginning with dissection of the uncinate process), and mesenteric approaches [4]. Each of these techniques presents unique advantages and limitations. Notably, the left-sided approach has been predominantly reported by Japanese surgical groups [6,7,8,9].

In 2021, Nagakawa et al. further reviewed the various approaches employed in minimally invasive PD. In their analysis, SMA approaches were classified into four primary types: anterior, posterior, right-sided, and left-sided approaches [10]. Most of the procedures described in their review were performed using laparoscopic techniques, and among the 23 articles referenced, only 3 involved robotic resections.

Currently, SMA dissection in RPD can be broadly categorized into two main approaches: right-sided and left-sided. Each of these includes several subtypes—such as the conventional, posterior, lateral, and anterior variants on the right side, and the normal and posterior variants on the left side. Table 1 summarizes the 27 articles identified through the scoping review process. In the following sections, each SMA approach is reviewed and discussed in detail, in alignment with the timeline presented in Table 1.

## 5. Right-Sided SMA Approach During RPD

### 5.1. Conventional Right-Sided Approach in RPD

Right-sided approach has been the most frequently reported and is considered the most conventional technique in RPD. In the early 2010s, the majority of RPDs were performed using the da Vinci S or da Vinci Si system, both of which had a fixed camera arm and a preoperatively designated traction arm positioned on either the right or left side [11,12,13,14,16,17]. Consequently, the robotic camera was typically fixed at the right abdomen or midline incision.

In 2010, Giolianotti et al. presented the world’s first comprehensive report on the technical aspects of RPD using da Vinci standard/S system [11]. Based on a single-surgeon experience with 50 RPD cases, the authors detailed operative techniques using a right-sided SMA approach with a right-sided scope. Their findings demonstrate the safety, feasibility, and advantages of the robotic approach, highlighting its role in transforming complex open surgeries into minimally invasive operations with comparable morbidity and mortality.

In 2011, Nguyen et al. described the so-called Pittsburgh-style RPD, which incorporated initial laparoscopic dissection combined with robotic retraction and dissection. In this approach, SMA dissection was performed from the right side, with the endoscope inserted near the midline position [12]. Similarly, in 2013, Boggi et al. demonstrated an RPD technique in which the camera was placed in the right abdomen, and SMA dissection was conducted from the right side [13]. The fixed right-sided view made it most practical to perform all procedures from the supracolic region and the right side of the SMA and superior mesenteric vein (SMV). This approach provided the largest working space on the right and cranial sides of the SMA while eliminating the need for specific retraction of the transverse colon.

In 2015, Chen et al. reported a series of 60 consecutive RPDs using a right-sided approach with the da Vinci S system [14]. Their study compared the safety and efficacy of RPD and OPD in 180 patients with borderline or malignant pancreatic diseases. While the RPD group had significantly longer operative times, it demonstrated advantages such as reduced blood loss, faster postoperative recovery, shorter hospital stays, and better postoperative nutritional status. Both groups showed comparable rates of surgical morbidity, mortality, R0 resection, and long-term oncologic outcomes. The study also highlighted a learning curve effect for RPD, reinforcing its feasibility as a minimally invasive alternative to OPD for selected patients.

In 2017, Liu et al. further detailed RPD using a right-sided approach with the midline camera position in the da Vinci Si system [16]. They compared RPD with laparoscopic pancreaticoduodenectomy (LPD) in 52 patients with periampullary neoplasms. The RPD group exhibited significantly shorter operative times (mean 387 vs. 442 min), reduced blood loss (219 vs. 334 mL), and shorter hospital stays (17 vs. 24 days). There were no significant differences in complication rates, mortality rates, R0 resection rates, or the number of harvested lymph nodes. These findings suggest that RPD is a more efficient and safer alternative to LPD in appropriately selected patients. Marino et al. and Goh et al. reviewed their initial series of eight RPDs performed using a right-sided approach, further contributing to the body of evidence supporting this technique [17,18].

Wei et al. described their novel method for RPD resection, named “G”-shaped approach, wherein the SMA was dissected from the right side [26]. This technique involved a systematic sequence of steps, emphasizing early gastric mobilization, hepatic artery suspension, mesenteric vein exposure, and uncinate process dissection. Sixty consecutive patients were analyzed using cumulative sum analysis. After 16 cases, significant reductions in operative time, blood loss, hospital stay, and complications such as bile leakage and delayed gastric emptying were observed, suggesting the technique can effectively shorten the RPD learning curve.

As outlined above, pioneering RPD series reported in the 2010s predominantly utilized a right-sided approach with a fixed midline or right-sided scope, a practice likely influenced by the limitations of earlier da Vinci systems, which featured a fixed camera arm and a preoperatively determined retraction arm position.

In the 2020s, several groups began reporting RPDs with right-sided approaches utilizing the da Vinci Xi system. Ikoma et al. and Torres et al. introduced and refined the technique of extracorporeal retraction of the SMV to enhance exposure of the SMA from the right side [19,30]. In their reports, SMA dissection was performed entirely from the right, with horizontal retraction of the pancreatic head toward the right lateral direction. Their approach involved SMA dissection from the right side while dynamically adjusting SMV retraction to optimize exposure. The endoscope was inserted via the right-sided port (R2 arm), facilitating an unobstructed, direct view of the right aspect of the SMA.

Zward et al. reported a detailed, standardized RPD technique for pancreatic head cancer in a patient with a replaced right hepatic artery. The procedure was performed using the da Vinci Xi system. Key technical steps included careful skeletonization and looping of the aberrant artery, portal dissection, pancreatic neck transection, and uncinate dissection along the SMA and SMV from the right side [27].

Mizumoto et al. analyzed 76 RPD cases to evaluate the learning curve and technique optimization, particularly the nerve plexus hanging maneuver under right-sided SMA approach [34]. Over time, operation time and major complications significantly decreased. The introduction of this maneuver contributed to safer dissection of the uncinate process, especially around the SMA/SMV, and facilitated training of new surgeons.

### 5.2. Right Posterior Approach in RPD

In 2016, Memeo et al. first described the SMA-first dissection in the field of RPD. In this case report, the procedure began with peritoneal exploration, followed by SMA dissection from the right posterior side using a hanging maneuver without dividing any other critical organs [15]. Machado et al. reported a retrospective analysis of RPD cases with the right posterior SMA-first approach in RPD for pancreatic and periampullary tumors [20]. Among 73 patients who underwent RPD, 24 received the SMA-first approach, including cases requiring venous resection. Similarly, Azagra et al., Bhanrare et al., and Mourthadhoi et al. reported a case video with hemi-circumferential SMA dissection (level-3 dissection) using a right posterior approach [21,33,35,37]. Based on these reports, the right posterior approach in RPD facilitates early and clear exposure of the SMA, enhances the quality of lymphadenectomy, and contributes to safer tumor resection by improving control of the posterior surgical margin.

### 5.3. Right Lateral Approach in RPD

Ninomiya et al. described a right-lateral approach to the SMA, involving vertical retraction of the pancreatic head [29]. By rotating the pancreaticoduodenum and exposing the SMA from its outer right surface, this technique facilitates dissection, reduces operative time, and minimizes blood loss. In a study involving nine patients, the median operative time was 329 min, and the median blood loss was 16.5 mL.

As described above, right-sided SMA approaches have long been the standard in RPD. These techniques were originally developed based on the ergonomics of the early da Vinci system and were subsequently refined into standardized variations—including the ordinal right, right posterior, and right lateral approaches—to accommodate more advanced dissection strategies for pancreatic cancer.

### 5.4. Extended Indications of Right-Sided Approaches in RPD

To achieve optimal oncological local control, several groups have demonstrated radical SMA dissection for invasive pancreatic cancer using peri-arterial divestment techniques. Kinny-Köster et al. introduced the so-called “triangle resection,” which was originally introduced during open pancreatoduodenectomy by the Heidelberg group, and its concept was subsequently applied to robotic procedures, which involve a hemi-circumferential dissection of the nerve plexus around the SMA (also referred to as “level 3 dissection”) via a right-sided approach [22,38]. Similarly, Shyr et al. reported surgical outcomes from a series of 289 RPD cases, including 36 cases with level 3 SMA dissection [23]. Their study assessed the feasibility of level 3 dissection in RPD, analyzing a total of 289 RPD and 162 OPD cases. In RPD, level 3 dissection was associated with a higher incidence of postoperative diarrhea (34.5%, *p* < 0.001) and greater blood loss (median 263 mL, *p* = 0.015). However, it resulted in a significantly higher R0 resection rate (>1 mm margin: 93.8% vs. 72.2%, *p* < 0.001) and an increased lymph node yield (*p* < 0.001) compared to lower-level dissections. Surgical mortality and major complication rates were comparable between groups. When compared to OPD, RPD with level 3 dissection demonstrated reduced blood loss, an absence of delayed gastric emptying, and a lower incidence of chyle leakage, suggesting that RPD is a safe and effective alternative for level 3 mesopancreas dissection. Kaufman et al. assessed the feasibility of “cold” triangle RPD for pancreatic ductal adenocarcinoma [25]. The technique involves sharp dissection around key vessels without energy devices using the right-sided SMA approach. Among 127 consecutive cases, triangle dissection was completed robotically in all, with a 2.4% conversion rate and a 22% severe complication rate. The 90-day mortality dropped to 2.3% after the learning curve. Cold triangle RPD demonstrated acceptable morbidity and enabled high-quality resection and staging, supporting its feasibility in selected patients.

## 6. Left-Sided SMA Approach During RPD

### 6.1. Left-Posterior Approach in RPD

In OPD, the left posterior approach has been one of the commonly utilized techniques to facilitate the SMA-first maneuver [6,7,8,9]. In this approach, the SMA is dissected from its left aspect along with an en bloc resection of the proximal mesojejunum and mesopancreas [39]. This technique is feasible in OPD because the open view of the infracolic space can be easily maintained using surgical retractors or the assistant’s hands.

However, in the RPD setting, two major technical challenges arise in implementing the left posterior approach. The first challenge is cranial retraction of the transverse colon. To dissect the SMA from the left side, surgical maneuvers must be performed within the infracolic space, necessitating continuous and thorough retraction of the transverse colon and mesocolon. If a robotic arm is dedicated to this retraction, all dissection must be performed using only the remaining two robotic arms, limiting operative flexibility. The second challenge is the viewing angle. As described in the early RPD series using the da Vinci S/Si system, the camera port was fixed at the midline or on the right side of the abdomen throughout the procedure. In this setup, the left side of the SMA is visualized at an oblique angle, making dissection technically unfavorable. Furthermore, dedicating one robotic arm to transverse colon retraction in this context seems impractical given the potential trade-offs in instrument functionality. Due to these challenges, no reports advocated for left-sided approaches in RPD during the da Vinci S/Si era.

**Figure 1:**
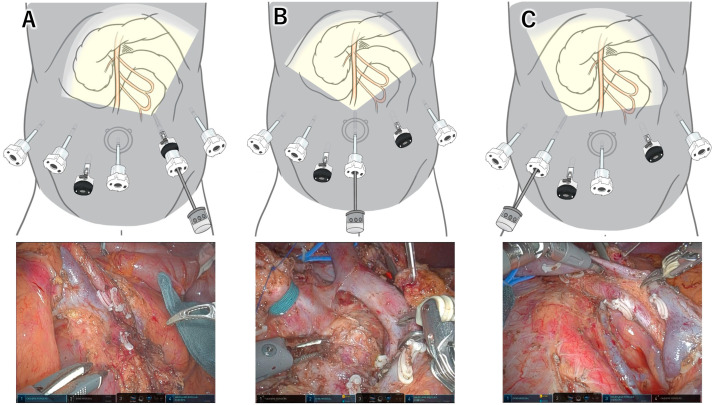
**Differences in the field of view provided by the scope transition method**. (**A**) The left-side position offers a direct, open view of the left-posterior aspect of the superior mesenteric artery (SMA). (**B**) The central position provides the widest field of view and is suitable for dividing critical structures and performing dissections along the celiac–hepatic arterial system. (**C**) The right-sided position is used during the final stage of resection, allowing access to the right aspect of the SMA. Reconstruction procedures are also performed from this position.

In 2022, Inoue et al. first introduced the scope transition (ST) method, which enables adjustment of the viewing angle during the resection phase of RPD [40]. This method allows for optimization of the visual perspective at each procedural step. In their report, they presented a case employing a novel robotic left posterior SMA-first approach, in which SMA dissection was conducted before the division of critical structures such as the pancreas, stomach, bile duct, and jejunum. A “left position” view, achieved by inserting the scope through a left abdominal port (Figure 1), provided direct visualization of the left aspect of the SMA. In addition to the enhanced viewing angle, a key technical challenge—sustained retraction of the transverse colon—was addressed using mechanical cephalad retraction with the Nathanson liver retractor. These two technical innovations enabled the successful implementation of the left posterior approach in the RPD setting.

The ST method comprises two principal techniques: redocking and port-hopping. Redocking entails repositioning the robotic arms to adjacent trocar sites. Specifically, during the transition from the left to the central position, robotic arms R2 and R3 are temporarily undocked and relocated from the midline and left-sided trocars to the right-sided and midline trocars, respectively. In contrast, port-hopping involves exchanging the endoscope and instruments without altering robotic arm positions. During port-hopping from the central to the right position in RPD, the endoscope is moved from R3 to R2, the traction arm from R1 to R4, the left-hand instrument from R2 to R1, and the right-hand instrument from R4 to R3. Kitano et al. further refined the ST method, demonstrating that redocking and port-hopping could be completed within 60 to 90 s following procedural optimization [41].

In 2024, Takagi et al. reported a RPD technique incorporating a left posterior approach to the SMA [31]. In their procedure, the so-called “two-surgeons technique” enabled a rapid and seamless dissection of the left aspect of the SMA. Following mobilization of the pancreatic head and division of the proximal jejunum, the mesopancreas was retracted rightward, allowing for the identification, dissection, and ligation of the relevant SMA branches from the left posterior view. Furthermore, a mesopancreas-hanging technique was employed to facilitate stable retraction of the mesopancreas during subsequent right-sided SMA dissection. Thus, their strategy represents a combined left- and right-sided approach for SMA dissection in RPD.

In 2025, Kiritani et al. reported a retrospective study evaluating the left posterior approach to the SMA in RPD [36]. Their findings suggested that initiating dissection from the left and posterior aspects of the SMA facilitated early vascular control and circumferential clearance. A retrospective analysis of 83 cases demonstrated improved procedural efficiency with increasing surgical experience. In the later phase of the study, operative times were significantly reduced, and the rate of early inferior pancreaticoduodenal artery (IPDA) ligation reached 90%. These findings indicate that the left posterior approach enhances surgical precision and may be established as a standard SMA-first technique for RPD.

### 6.2. Normal Left-Sided Approaches in RPD

In 2022, Takagi et al. described a left-sided approach to the SMA as an alternative technique for SMA dissection, highlighting its potential role within the broader surgical strategy for RPD [24]. While the right-sided approach is commonly utilized, it may be suboptimal in patients with obesity, dense adhesions, or malignancies requiring extensive lymphadenectomy. The authors proposed a novel combined left-and-right approach, which facilitated circumferential dissection of the SMA and ensured comprehensive lymph node clearance. Their findings emphasized the importance of tailoring the surgical approach based on individual anatomical and pathological considerations to optimize patient outcomes.

In 2023, Nakata et al. introduced a left-sided approach for mobilization of the pancreatic head during both LPD and RPD, with particular emphasis on complete dissection of the ligament of Treitz [28]. By exposing the SMA and jejunal arteries (JAs) from the left side, this technique enhanced pancreatic head mobilization and improved intraoperative visibility. An analysis of 75 cases demonstrated the safety of this approach, with reduced intraoperative blood loss and no procedure-related mortality. In contrast to the technique described by Inoue et al., Nakata et al. did not utilize ST to achieve a left-sided view. Rather, their left-sided approach to the SMA served primarily as a preparatory maneuver for subsequent right-sided SMA dissection. This technique provided optimal counter-traction between the pancreatic head and the SMA during right-sided dissection, conceptually aligning with Takagi et al.’s proposed left-and-right combined approach [24].

### 6.3. Extended Indications of Left-Sided Approaches in RPD

To achieve negative surgical margins in cases of invasive pancreatic head tumors, aggressive vascular dissection or resection is occasionally required. Some reports about left-sided approaches have proposed techniques for perivascular dissection for advanced pancreatic cancers.

Omiya et al. described LV-3 SMA dissection, involving a left-sided approach to the SMA to achieve R0 resection in borderline resectable pancreatic cancer [42]. Utilizing a left posterior approach, optimal countertraction between the SMA and the pancreatic head was achieved, facilitating safe and precise hemi-circumferential periarterial divestment. Importantly, LV-3 dissection was performed before the division of any critical structures such as the pancreas or bile duct, representing a true SMA-first strategy, akin to those described in prior reports on OPD.

Kobayashi et al. reported a series of portal vein resections and reconstructions performed during RPD. Employing a left posterior approach, they accessed the SMA first without requiring retraction of the SMV [43]. Given the anatomical relationship in which the SMA lies to the left of the SMV, the left-sided approach enabled earlier exposure of the SMA compared to the SMV. Following completion of oncological SMA dissection, the SMV was approached from the left posterior aspect, and a tumor-free segment was encircled. Subsequently, division of critical structures—such as the pancreas, bile duct, and stomach—was performed, along with en bloc dissection of suprapancreatic and hilar lymph nodes. The tumor was thereby completely isolated, except for its attachment to the SMV. As demonstrated in their series, the left posterior approach facilitated safe portal vein resection, with a median blood loss of 95 mL and no major postoperative complications among four patients.

## 7. Other SMA Approach During RPD

In 2023, Takagi et al. reported a case of RPD utilizing the anterior SMA-first approach for pancreatic cancer [32]. A 75-year-old man with a resectable tumor involving the SMA and SMV underwent successful RPD following neoadjuvant chemotherapy. The anterior SMA approach allowed for circumferential dissection of the artery and safe tumor removal without the need for vascular reconstruction. Notably, the authors also dissected the left posterior aspect of the SMA via the infracolic space to secure the posterior margin and to divide the ligament of Treitz. This maneuver facilitated optimal countertraction between the pancreatic head and the SMA. As such, the report effectively described a combined anterior and left-sided approach to enable complete circumferential dissection around the SMA. The patient achieved an R0 resection and experienced an uneventful postoperative recovery.

## 8. Technical Issues Associated with Right- and Left-Sided Approaches

### 8.1. Strengths and Limitations of the Right-Sided Approach

The right-sided approach remains the conventional and currently standard method for SMA dissection during RPD. This approach has two primary advantages. First, it provides simultaneous access to both the SMA and the celiac–hepatic arterial system, which is another critical target for dissection [44]. Typically, the right-sided approach does not require ST to reach both arterial territories. Second, it avoids the need for transverse colon retraction, preserving a relatively spacious working area in the right abdomen. In most cases, SMA dissection is performed from the right and supracolic perspective, without the need to visualize or manipulate the left aspect of the SMA.

**Figure 2:**
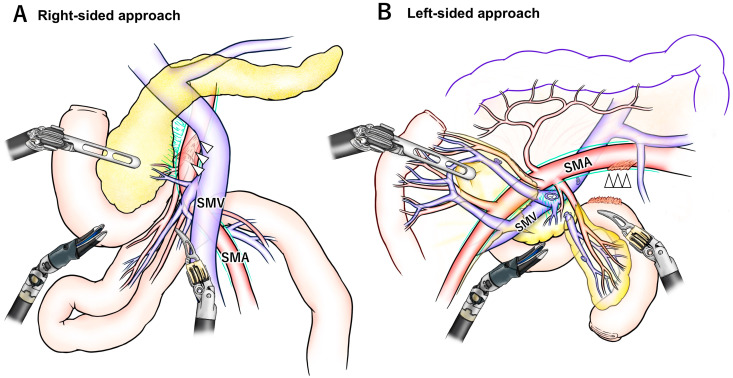
**Comparison between the right-sided approach and the left-sided approach**. (**A**) The right-sided approach does not require cephalad retraction of the transverse colon. Following the mobilization and extraction of the proximal mesojejunum, the jejunal loop assumes a distorted “β”-shaped configuration, as the jejunal origin is suspended cranially by the ligament of Treitz (white arrowheads). (**B**) In contrast, the left-sided approach necessitates cephalad retraction of the transverse colon. The proximal jejunal loop remains in its original in situ position, and the vessels supplying the pancreatic head and the mesojejunum planned for resection are stretched radially in a “wing-shaped” fashion. The ligament of Treitz (white arrowheads) can be divided at any desired time during the procedure. SMA, superior mesenteric artery; SMV, superior mesenteric vein.

**Figure 3:**
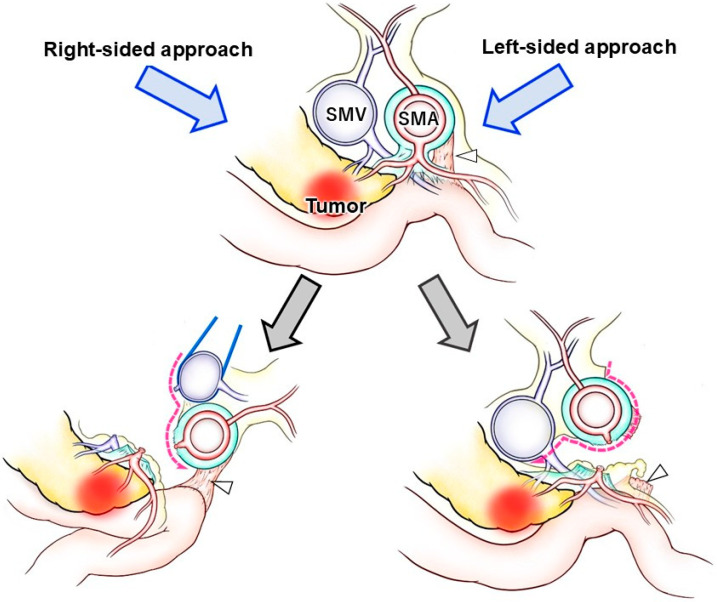
**Comparison between the right-sided approach and the left-sided approach (axial view)**. In the right-sided approach, retraction of the SMV is required to dissect around the SMA (dashed arrows). Because the jejunal origin is cranially suspended by the ligament of Treitz (white arrowheads), the proximal jejunum becomes twisted. In contrast, in the left-sided approach, the proximal jejunal loop remains in its natural in situ position, allowing direct dissection around the SMA from the left side without SMV retraction. The ligament of Treitz can be divided at any desired stage of the procedure. SMA, superior mesenteric artery; SMV, superior mesenteric vein.

However, the right-sided approach has certain limitations. One of the key challenges lies in the complex anatomy surrounding the uncinate process, a crucial area for achieving adequate SMA dissection. As illustrated in Figure 2, the right- and left-sided approaches show notable differences in the orientation of the mesojejunum and exposure to the SMA, which influence the sequence of dissection. In PD, the proximal jejunum must be resected along with the duodenum. Moreover, to achieve oncological clearance of the posterior SMA margin, thorough dissection of the soft tissue and lymph nodes posterior to the SMA is necessary. To facilitate this, an en bloc resection of the mesojejunum, including the common trunk of the JA and IPDA, is a reasonable strategy. During the right-sided approach, it requires the proximal mesojejunum is mobilized and passed to the right of the SMA via a retro-SMA route. However, this maneuver is often impeded by cephalad suspension of the proximal jejunum at the ligament of Treitz, resulting in a distorted, “β”-shaped configuration of the extracted jejunal loop (Figure 2A and Figure 3).

One commonly adopted workaround is dissection along the jejunal wall while preserving the mesojejunum. Although widely practiced in the right-sided approach, this method leaves the jejunal vessels and associated mesojejunum intact, thereby potentially compromising the certainty of a negative SMA margin in a case of invasive uncinate tumor. For non-invasive periampullary tumors or early-stage cancers with minimal risk of lymph node metastasis near the SMA, this approach may be justified as a safe and expedient technique.

Another proposed solution is extensive mobilization of the hepatic flexure of the colon and derotation of the mesentery, as described in reports of LPD [45]. However, even with this technique, the mesojejunum often remains distorted and twisted, which complicates precise identification of the intended transection plane necessary to include the common trunk of the JA and IPDA within the resection (Figure 2A).

An additional drawback of the right-sided approach is the potential presence of unexpected adhesions in the infracolic space. In such cases, it becomes difficult or impossible to extract the jejunum into the right supracolic region, necessitating additional adhesiolysis in the left infracolic area.

### 8.2. Strengths and Limitations of the Left-Sided Approach

The advantages of the left-sided approach can be categorized into three main aspects. First, it preserves the in situ orientation of the SMA/SMV, their relevant branches, the mesojejunum, and the uncinate process. Unlike other approaches, there is no significant distortion or torsion of the SMA/SMV pedicle, and each branch can be retracted in a “wing-shaped” fashion, which facilitates precise identification of the branches to be ligated or preserved [36]. As a result, peri-SMA dissection can be performed accurately in accordance with preoperative planning or by referencing CT findings intraoperatively. Additionally, the mesojejunum, which lies in a flattened and expanded configuration, contains radially branching JAs and jejunal veins (Figure 2B). This anatomy supports safe, bloodless dissection of the mesojejunum, particularly with awareness of key vascular landmarks such as the “dangerous crossover vein” and the inferior pancreatoduodenal veins [46]. Second, the ST method provides an open and direct view of the dorsal aspect of the SMA. This left-dorsal visual angle is unique, enabling dissection between the uncinate process and the SMA to be carried out directly in front of the surgeon’s field of view, without the need for oblique or tangential visualization (Figure 2B). Third, the left-sided approach allows for early access to the SMA before encountering the SMV. In the robotic setting, where only three working arms are available, this sequence is advantageous. Because the SMA consistently lies to the left of the SMV, dissection of the SMA can be accomplished without the need for SMV retraction using a tape or a traction arm. This allows the traction arm to be repurposed for other maneuvers (Figure 3).

In the standard right-sided approach, dissection of the superior mesenteric artery (SMA) requires mobilization of the proximal jejunum to the right side of the SMA through the retro-SMA pathway. Furthermore, dissection and retraction of the superior mesenteric vein (SMV) are essential to achieve adequate exposure of the SMA. In contrast, the left-sided approach enables early access to the SMA, allowing its complete dissection without the need for SMV retraction. The ligament of Treitz (white arrowhead) is deeply located and difficult to divide during the initial phase of the right-sided approach, whereas in the left-sided approach, it can be easily divided at any desired timing.

Despite these advantages, the left-sided approach has several limitations. First, it requires the creation of an optimal viewing angle toward the SMA. In a setting similar to the right-sided approach, the left side of the SMA may be visualized only tangentially, and its dorsal aspect may not be accessible. This can hinder safe dissection and ligation of key branches such as the common trunk of the JA and IPDA, or tumor-relevant JAs, particularly when these branches originate from the left-dorsal side (3–6 o’clock position) of the SMA. The ST method helps overcome this drawback by providing a straight-on view of the SMA’s left aspect. Furthermore, bidirectional retraction of the future-remnant and future-resected mesojejunum enhances exposure of the SMA’s dorsal surface [36,42,43]. Second, cranial retraction of the transverse colon is required to access the left side of the SMA in the infracolic space. Although this can be theoretically and practically achieved using a traction arm, occupying the traction arm for this purpose limits its availability during critical phases such as peri-SMA dissection. In such cases, the procedure must proceed with only two working arms, which can compromise counter-traction or rapid response to unexpected bleeding. To address this, mechanical retraction of the transverse colon using a Nathanson liver retractor may be a preferable strategy, freeing the traction arm to assist the surgeon’s hands more effectively [36,40]. Third, the left-sided approach remains relatively underutilized in RPD. Although the limited literature is currently available, reports on this technique are rapidly increasing, particularly from Japanese surgical groups, where the left-posterior approach is already a commonly adopted SMA dissection strategy in OPD. 

## 9. Future Perspectives

As described above, each approach to the SMA during PD presents unique advantages and limitations. The anatomical relationship between the pancreatic head and the SMA is highly complex due to variations in arterial branching patterns, venous anatomy, tumor location, extent of resection, and mesenteric rotation. The right-sided approach has traditionally been considered ergonomic and compatible with the original da Vinci surgical system. In contrast, the left-sided approach offers advantages in terms of anatomical naturality and alignment.

Unlike OPD, in which the choice of SMA approach has historically depended on individual surgeon preferences or philosophies, RPD techniques have evolved under the constraints of robotic technology. With the advent of the da Vinci Xi system, which offers enhanced flexibility and multi-quadrant access, the left-posterior approach has gained traction and been rapidly developed. Nonetheless, specific technical tips remain essential to perform left-sided SMA dissection comfortably and safely.

Future advancements in robotic platforms are expected to further overcome the current limitations of both right- and left-sided approaches. For instance, a robotic scope with a movable head may eliminate the need for ST to achieve optimal viewing angles. The addition of a fifth robotic arm, specifically designed for cranial retraction, could obviate the need for mechanical devices and free up the three primary working arms other than the camera arm. In parallel, a more comprehensive understanding of the detailed anatomy and procedural steps for SMA dissection will help surgeons tailor the approach, sequence, and extent of dissection based on tumor location and oncologic requirements. A flexible, combined application of both approaches may ultimately enable safer and more advanced dissection, even in complex cases such as borderline resectable pancreatic cancers.

Overall, the benefits and limitations of each SMA approach should be evaluated through prospective or rigorously designed retrospective comparative studies. Although some randomized controlled trials have demonstrated the superiority or non-inferiority of RPD compared to OPD in terms of short-term outcomes [47,48], these studies have generally employed multi-center designs without standardized surgical protocols, particularly regarding SMA dissection techniques. Consequently, no current study has directly compared the outcomes of different SMA approaches within RPD. Once each approach has reached sufficient technical maturity, prospective comparative trials will be warranted to determine the optimal SMA dissection strategy in the robotic era.

## 10. Limitations

This scoping review has several limitations. First, it is inherently limited by the nature of the included studies, which were mostly retrospective, single-institutional reports or case series. Consequently, the level of evidence remains low, and direct comparison between different SMA dissection approaches is not feasible. Second, oncological and perioperative outcomes associated with each approach were not systematically compared, as many studies focused primarily on technical descriptions. Third, while the current review focuses on procedures performed using the da Vinci robotic systems, it does not address alternative or emerging robotic platforms. As such, the findings may not be generalizable to other robotic technologies that differ in ergonomics, instrumentation, or visualization. Fourth, publication bias may exist, as technically challenging or unsuccessful approaches may be underreported. Lastly, while our review was conducted based on a comprehensive search strategy, relevant studies indexed in other databases or published in languages other than English might have been missed.

## 11. Conclusions

This review has discussed the recent advancements and current status of SMA dissection in the context of RPD. The evolution of robotic technology has expanded the available strategies for the SMA approach, with the left-sided technique emerging as a promising alternative due to its anatomical precision. Further refinements in surgical technique, a deeper understanding of mesenteric anatomy, and continued development of robotic platforms will contribute to more precise, efficient, and safer SMA dissection in the future.

## Figures and Tables

**Table 1:** List of previous studies describing SMA approach.

Author	Year	Article Type	N	da Vinci Type	SMA Approach	Combination	Scope Position
Giulianotti [11]	2010	Retrospective analysis	50	Standard/S	Right	ND	Right
Nguyen [12]	2011	Case report	1	S	Right	ND	Midline
Boggi [13]	2013	Retrospective analysis	34	S/Si	Right	ND	Right
Chen [14]	2015	Retrospective analysis	60	S	Right	ND	Midline
Memeo [15]	2016	Case report	1	Si	Right posterior	ND	Midline/right
Liu [16]	2017	Retrospective analysis	27	S	Right	ND	Midline
Marino [17]	2018	Retrospective analysis	8	Si	Right	ND	Midline
Goh [18]	2019	Retrospective analysis	8	Si	Right	ND	Midline
Ikoma [19]	2020	Case report	1	Xi	Right	ND	Right
Machado [20]	2021	Retrospective analysis	73	Si/Xi	Right posterior	ND	Right
Azagra [21]	2021	Case report	1	Xi	Right posterior	ND	Right
Kinny-Köster [22]	2021	Case report	3	Xi	Right	ND	Right
Shyr [23]	2021	Retrospective analysis	289	Si/Xi	Right	ND	Midline/Right
Inoue [22]	2022	Retrospective analysis	14	Xi	Left posterior	Left & right	Left/Midline/Right
Takagi [24]	2022	Case report	1	Si	Left	Left & right	Right
Kaufman [25]	2022	Retrospective analysis	127	Si/Xi	Right posterior	ND	Right
Wei [26]	2022	Retrospective analysis	60	Si	Right	ND	Midline
Zwart [27]	2022	Case report	1	Xi	Right	ND	Right
Nakata [28]	2023	Retrospective analysis	43	Xi	Left	Left & right	Midline
Ninomiya [29]	2023	Retrospective analysis	9	Xi	Right lateral	ND	Right
Torres [30]	2024	Case report	1	Xi	Right	ND	Right
Takagi [31]	2024	Case report	1	Xi	Left posterior	Left & right	Midline
Takagi [32]	2024	Case report	1	Xi	Anterior	Left & Anterior	Midline
Bhandare [33]	2024	Case report	1	Xi	Right posterior	ND	Right
Mizumoto [34]	2024	Retrospective analysis	73	Si/Xi	Right	ND	Midline
Mourthadhoi [35]	2025	Case report	1	Xi	Right posterior	ND	Right
Kiritani [36]	2025	Retrospective analysis	83	Xi	Left posterior	Left & right	Left/Midline/Right

SMA, superior mesenteric artery; ND, not described.

## Supplementary Materials

The following supporting information can be downloaded at: https://www.mdpi.com/article/10.3390/jcm14176084/s1, Figure S1: PRISMA-ScR Flow Diagram for Study Selection; Table S1: PRISMA-ScR Checklist.

## Abbreviations

The following abbreviations are used in this manuscript:

PD	pancreaticoduodenectomy
RPD	robotic pancreaticoduodenectomy
SMA	superior mesenteric artery
OPD	open pancreaticoduodenectomy
SMV	superior mesenteric vein
LPD	laparoscopic pancreaticoduodenectomy
ST	scope transition
JA	jejunal artery
IPDA	inferior pancreaticoduodenal artery
