# Supplementary material for: Current Status and Future Perspectives of Superior Mesenteric Artery Dissection in Robotic Pancreaticoduodenectomy: A Scoping Review of Technical Variations in the Robotic Era
Source: J Clin Med. 2025 Aug 28;14(17):6084. doi: 10.3390/jcm14176084 (PMC12428843; doi:10.3390/jcm14176084)
Supplement: Supplementary file 1 [file jcm-14-06084-s001.zip › JCM-3614435-supplementary-Table S1.pdf]

Supplementary Table S1. PRISMA-ScR Checklist

SECTION	ITEM	PRISMA-ScR Checklist Item	REPORTED ON PAGE
TITLE	1	Identify the report as a scoping review.	Title page
ABSTRACT	2	Structured summary including background, objectives, eligibility criteria, sources of evidence, charting methods, results, and conclusions.	Page 1
INTRODUCTION	3	Describe the rationale for the review in the context of what is already known.	Page 2
	4	Provide an explicit statement of the questions and objectives being addressed.	Page 2
METHODS	5	Indicate whether a review protocol exists, and if so, where it can be accessed.	Not applicable (no protocol registered)
	6	Specify eligibility criteria.	Page 3
	7	Describe all information sources (e.g., databases with dates of coverage).	Page 2
	8	Present full electronic search strategy for at least one database, including limits used.	Page 3
	9	State the process for selecting sources of evidence.	Page 3
	10	Describe the methods of data extraction from included sources.	Page 3

	11	List and define all variables for which data were sought and describe any assumptions and simplifications made.	Page 3
	12	If done, describe any methods used to assess the quality of individual sources of evidence.	Not applicable
	13	Describe the methods of handling and summarizing the data.	Page 3
RESULTS	14	Provide numbers of sources of evidence screened, assessed for eligibility, and included in the review, with reasons for exclusions.	Page 4, Figure 1
	15	Present characteristics of included sources of evidence.	Page 4, Table 1
	16	If done, present data on the quality of included sources of evidence.	Not applicable
	17	Present the relevant results that relate to the review questions and objectives.	Page 4
DISCUSSION	18	Summarize the main results including an overview of concepts, themes, and types of evidence available.	Page 4 - 11
	19	Discuss limitations of the scoping review process.	Page 14
	20	Provide a general interpretation of the results and	Page 11 - 14

		implications for future research.	
FUNDING	21	Describe sources of funding for the included sources of evidence, and for the scoping review itself.	Page 15
