# Supplementary figures and images for: Current Status and Future Perspectives of Superior Mesenteric Artery Dissection in Robotic Pancreaticoduodenectomy: A Scoping Review of Technical Variations in the Robotic Era
Source: J Clin Med. 2025 Aug 28;14(17):6084. doi: 10.3390/jcm14176084 (PMC12428843; doi:10.3390/jcm14176084)

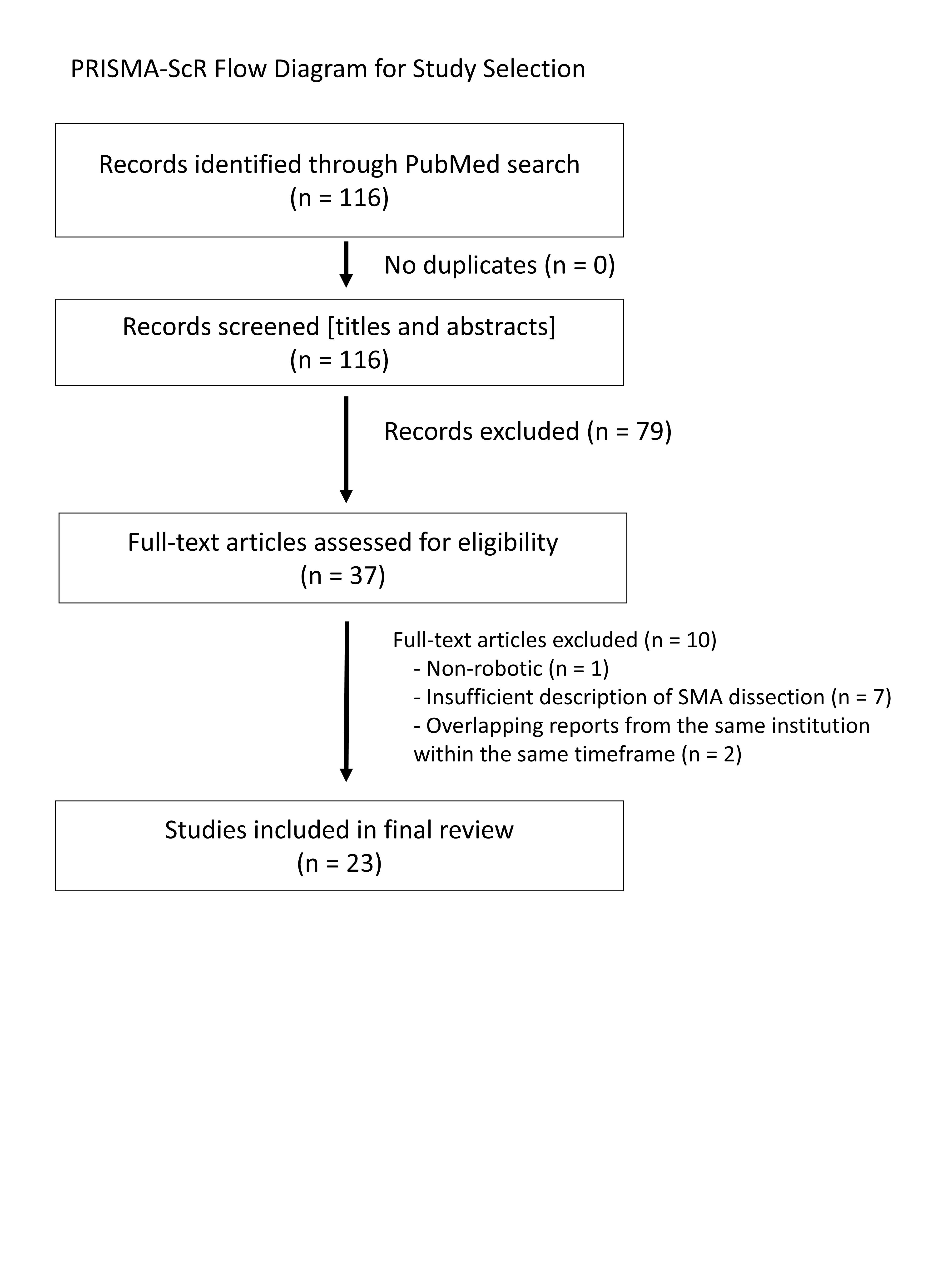

Supplement: Supplementary file 1 [file jcm-14-06084-s001.zip › jcm-3614435-supplementary-Figure S1.jpg]
